# TransCat: a hybrid CNN-transformer network with KAN for medical image segmentation

**DOI:** 10.3389/fbinf.2026.1849059

**Published:** 2026-08-21

**Authors:** Jin Wang, Zhenghua Yang, Dongming Zhou, Gang Chen, Sihan Wang, Yuhong Liu

**Affiliations:** 1 School of Electronic Science and Engineering, Hunan University of Information Technology, Changsha, China; 2 School of Information Science and Engineering, Yunnan University, Kunming, China

**Keywords:** CNN–Transformer, deformable attention, intelligent sensing, Kolmogorov–Arnold Network, medical image segmentation, multi-scale global modeling

## Abstract

Medical image segmentation is a fundamental task in computer vision and plays an important role in clinical diagnosis and treatment planning. Existing methods are mainly built on either convolutional neural networks (CNNs) or Transformer-based encoder-decoder architectures. CNNs are effective at capturing local patterns, whereas Transformers are better suited to modeling long-range dependencies. However, current hybrid designs still face three persistent challenges: insufficient exploitation of multi-scale CNN features for global modeling, rapidly increasing attention cost after token expansion, and ineffective fusion between CNN and Transformer representations. To address these issues, we propose TransCat, a hybrid CNN-Transformer architecture for medical image segmentation. First, we introduce Patch Concat, which converts multi-scale CNN features into patch tokens and feeds them jointly into the Transformer, enabling global reasoning across multiple scales. Second, to control the computational burden caused by the enlarged token set, we develop an extended deformable attention mechanism with attentive value identification. Third, we design a Kolmogorov–Arnold Network (KAN) attention module for adaptive cross-branch fusion. The module projects high-level CNN and Transformer features into a shared space and uses a KAN layer to estimate data-dependent pixel-wise weights. These weights selectively modulate the globally modeled Transformer features, allowing complementary local structural evidence from the CNN branch to be incorporated according to the image content rather than through fixed addition or concatenation. Experiments on multiple medical image segmentation benchmarks show that TransCat is competitive under the reported protocols, with its clearest advantage observed on unseen polyp test data.

## 1 Introduction

Medical image segmentation is a core task in computer vision and plays an important role in clinical diagnosis and treatment planning. With the rapid growth of computing power, deep learning-based segmentation methods have gradually replaced earlier approaches that relied heavily on hand-crafted priors (Cheng, 1995; Pun, 1980; Tremeau and Borel, 1997). Current deep medical image segmentation models can be broadly divided into two categories according to their underlying architectures: convolutional neural network (CNN)-based methods and Transformer-based methods.

CNN-based models have achieved remarkable success in medical image segmentation, especially since the introduction of the U-Net encoder-decoder architecture (Ronneberger et al., 2015). Although CNNs are highly effective at extracting local details, their ability to capture long-range contextual information is limited. Attempts to alleviate this limitation through attention mechanisms or redesigned skip connections (Zhou et al., 2018; Oktay et al., 2018; Jin et al., 2020) still inherit the intrinsic locality imposed by fixed receptive fields.

Transformers were originally proposed for sequence modeling in natural language processing (Vaswani et al., 2017) and were later introduced into computer vision through Vision Transformers (ViTs) (Dosovitskiy et al., 2020). Their main advantage lies in modeling global dependencies through direct interactions among tokens. However, when applied to medical image analysis, Transformers often struggle to preserve fine-grained local details. Even variants that incorporate convolutional inductive biases Wu et al. (2021) still face challenges in balancing global modeling and precise local representation. Transformer-based segmentation architectures such as SegFormer have also demonstrated the effectiveness of global token modeling (Xie et al., 2021).

As the complementary strengths and weaknesses of CNNs and Transformers have become better understood (Liu et al., 2021; Wang et al., 2023a), research has increasingly shifted from improving each paradigm independently to integrating them more effectively. As a result, a large number of hybrid architectures have been proposed. Nevertheless, most mainstream medical image segmentation networks still follow the U-Net-style encoder-decoder paradigm, in which the encoder extracts increasingly abstract semantic features through progressive downsampling and the decoder gradually reconstructs the target segmentation map. During this process, however, detailed spatial information is often weakened or lost, and the resulting hybrid designs still face three related bottlenecks: how to preserve multi-scale details for global modeling, how to keep attention computation tractable after token expansion, and how to fuse CNN and Transformer features without introducing additional information loss. The fusion problem is not merely a matter of combining two feature tensors. CNN features primarily preserve local texture and boundary cues, whereas Transformer features encode broader semantic dependencies; moreover, their relative importance can vary across target interiors, ambiguous boundaries, small objects, and background regions. Fixed operations such as addition or concatenation, when used alone, do not explicitly model spatially varying cross-branch interactions and may therefore transmit irrelevant local responses or underuse complementary structural information.

**FIGURE 1:**
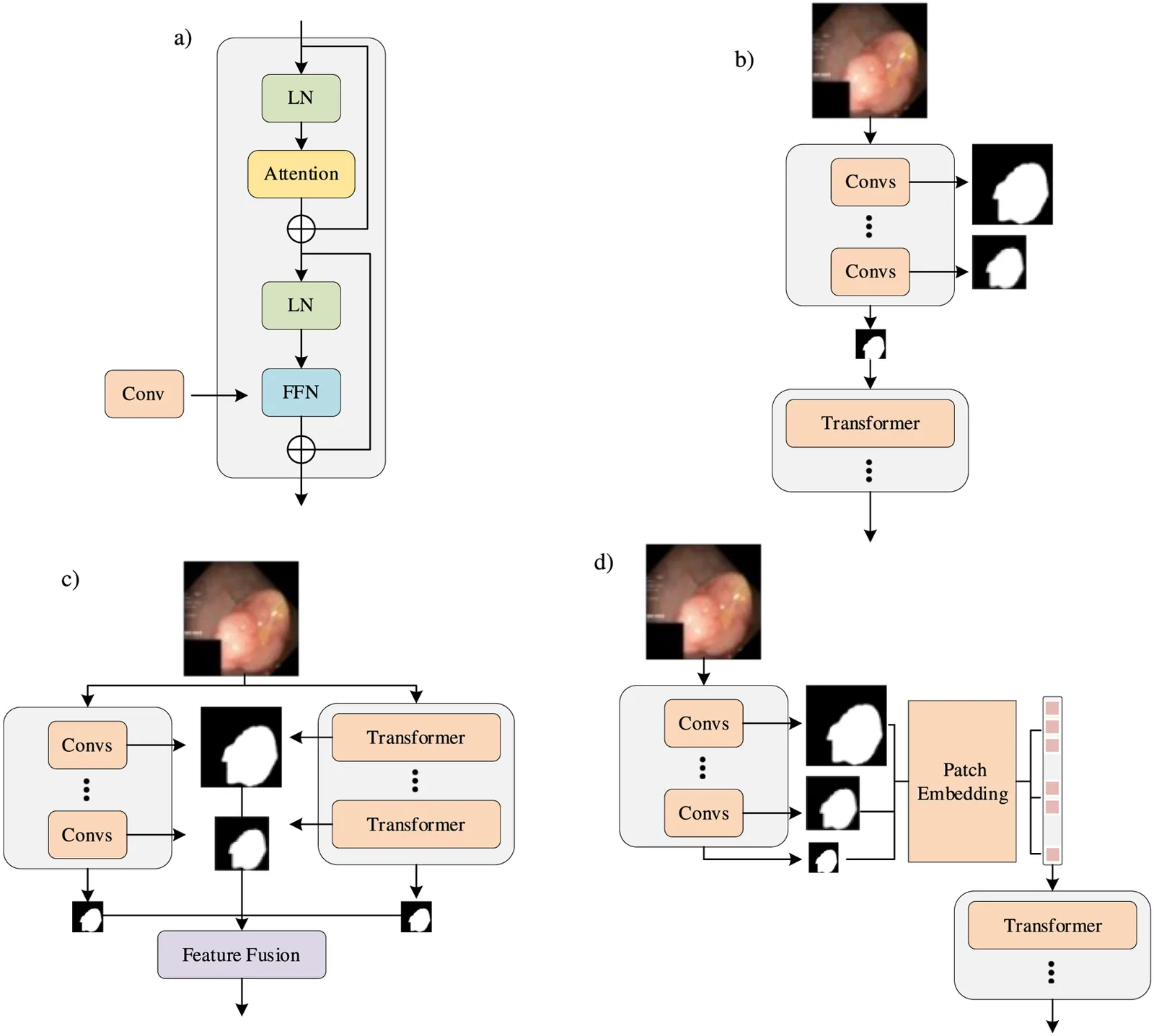
Representative strategies for integrating CNNs and Transformers in medical image segmentation: **(a)** framework embedding, **(b)** hierarchical stacking, **(c)** parallel processing, and **(d)** the proposed TransCat design.

The mainstream methods for integrating CNNs and Transformers can be broadly categorized into three approaches, as illustrated in Figure 1: (1) *Embedding convolution into the Transformer framework*. As shown in Figure 1a, this strategy inserts depth-wise convolution into the feed-forward layer of the Transformer to enhance local feature extraction (Huang et al., 2023). (2) *Hierarchical stacking*. As shown in Figure 1b, this approach uses a CNN to extract feature representations before subsequent Transformer-based global encoding, as exemplified by TransUNet Chen et al., 2021. In most cases, shallow CNN features are used during upsampling as complementary information, whereas high-level features are used for global context modeling. However, shallow features often contain critical boundary cues, and relying only on single-scale high-level features for global modeling may discard useful edge information. (3) *Parallel processing*. As shown in Figure 1c, this strategy places a shallow CNN encoder and a Transformer branch in parallel and fuses their features afterward (Zhang et al., 2021; Sanderson and Matuszewski, 2022). Although such designs can capture both local and global context through multi-scale representations, determining an effective fusion strategy remains difficult. Existing choices, including addition, concatenation, and channel attention (Qin et al., 2020; Cui et al., 2023), each have their own limitations. Figure 1d presents our backbone design. By reshaping the multi-scale features extracted by the CNN into 16 × 16 patches and concatenating the resulting tokens, our method performs global modeling across multiple scales without requiring an additional pre-fusion stage. Cross-scale boundary modeling has also been investigated to preserve fine-grained structures in medical image segmentation (Wang et al., 2023b).

Motivated by these limitations, this study aims to develop a hybrid architecture in which the roles of the CNN and Transformer are more clearly organized: CNN features provide rich multi-scale local evidence, the Transformer performs global reasoning over those features, and the fusion module selectively combines the complementary information from both branches. The architecture follows a direct problem-to-module rationale. Patch Concat preserves multi-scale CNN evidence before global reasoning; the extended deformable attention mechanism controls the attention cost introduced by the enlarged token set; and KAN attention provides data-dependent cross-branch fusion. Specifically, Patch Concat transforms the multi-scale features extracted by ResNet50 into patch tokens and feeds them jointly into the Transformer (He et al., 2016), allowing global reasoning to operate on multiple spatial scales rather than on a single high-level feature map. Attentive value identification is then incorporated into deformable attention to aggregate informative sampled values without applying dense attention to all concatenated tokens. Finally, the proposed KAN attention module (Liu et al., 2024) projects high-level CNN and Transformer features into a shared space and estimates pixel-wise fusion responses. After sigmoid activation, these responses modulate the Transformer representation, enabling local structural evidence to be incorporated selectively according to the joint feature content. KAN attention therefore serves as an adaptive fusion function rather than a replacement for either backbone branch. Accordingly, the novelty of TransCat lies primarily in how these components are coordinated for medical image segmentation, rather than in claiming that patch tokenization, deformable attention, or KANs are individually new. In summary, our contributions are as follows:

- We propose TransCat as a coordinated hybrid segmentation framework in which multi-scale CNN features are exposed to Transformer-based global reasoning before cross-branch fusion.
- We introduce Patch Concat together with an extended deformable attention mechanism so that the enlarged multi-scale token set can be processed with targeted sampling rather than dense attention over all tokens.
- We design a KAN-based attention module that learns pixel-wise fusion responses from paired high-level CNN and Transformer features, thereby replacing fixed cross-branch mixing with content-dependent modulation.
- Experiments and ablation studies show that TransCat is competitive across several medical image segmentation benchmarks, with its clearest advantage appearing in cross-dataset polyp evaluation. Case-level bootstrap intervals are also reported for the fixed TransCat polyp predictions to provide an estimate of test-set uncertainty. Pyramid-pooling strategies have also been investigated for polyp segmentation (Hu et al., 2023).

## 2 Related works

### 2.1 CNN-, transformer-, and hybrid segmentation networks

Since the seminal work of Ronneberger et al. (2015), U-Net and its variants have remained central to medical image segmentation because their encoder-decoder structures and skip connections preserve local detail effectively. Recent studies have also explored diffusion-based enhancement, lightweight U-shaped networks, and spatial-pyramid attention for medical image segmentation (Dong et al., 2024; Feng et al., 2024; Hu et al., 2024). Attention U-Net (Oktay et al., 2018), U-Net++ (Zhou et al., 2018), and Duck-Net (Dumitru et al., 2023) further improve local feature selection, semantic alignment, or convolutional block design. Operators such as depth-wise convolution (Chollet, 2017) and deformable convolution (Dai et al., 2017) also improve computational efficiency or geometric flexibility. These CNN-based approaches are effective for boundary detail, but their interactions remain largely local and often require deeper encoders or elaborate fusion paths to approximate long-range reasoning.

Transformer-based and hybrid models address this limitation by introducing token-level global interaction. TransUnet (Chen et al., 2021) combines CNN feature extraction with Transformer encoding, TransFuse (Zhang et al., 2021) adopts parallel CNN and Transformer branches, and pyramid-style Transformer models such as PVT (Wang et al., 2021), SSFormer (Wang et al., 2022a), and FCBFormer (Sanderson and Matuszewski, 2022) introduce hierarchical representations for dense prediction. PVT v2 further improves the efficiency and representation capability of pyramid vision Transformers (Wang et al., 2022b). More recent segmentation networks have also explored multi-scale or adaptive receptive-field designs, such as MEGANet (Bui et al., 2024), MIRA (Yan et al., 2025), PARF-Net (Ma et al., 2025), and ScaleFusionNet (Qamar et al., 2025). These recent studies indicate that multi-scale representation, adaptive receptive fields, and scale-aware fusion remain active research directions. However, many of these designs either add convolutional locality to Transformer blocks, pass only limited CNN features to the global modeling stage, or fuse branch outputs after largely separate encoding. TransCat differs by feeding multi-scale CNN features into the Transformer through Patch Concat before cross-branch fusion, so the global reasoning stage receives richer multi-scale evidence rather than a single high-level feature map. Efficient CNN-based architectures, such as ConvUNeXt, have also been developed for medical image segmentation (Han et al., 2022).

### 2.2 Multi-scale feature fusion and KAN-based attention

Multi-scale feature fusion is usually implemented through addition, concatenation, channel attention, spatial attention, or cross-branch gating. These strategies are useful but can be either static or weakly adaptive when the relative importance of CNN and Transformer features varies across anatomical regions, lesion boundaries, and background areas. In this work, KAN attention is used as an adaptive high-level fusion function rather than as a replacement for the entire segmentation backbone. KANs Liu et al. (2024), inspired by the Kolmogorov-Arnold representation theorem, replace fixed activations with learnable univariate functions. We use this property to estimate data-dependent fusion responses from high-level CNN and Transformer features. Therefore, the claimed gap is not that existing fusion operators are ineffective in general, but that TransCat organizes multi-scale tokenization, targeted deformable aggregation, and adaptive high-level fusion in one medical segmentation pipeline.

## 3 Materials and methods

This section first presents the overall architecture of TransCat, then describes the patch concatenation strategy and the corresponding extension of deformable attention, and finally introduces the proposed KAN attention module.

### 3.1 Model architecture

**FIGURE 2:**
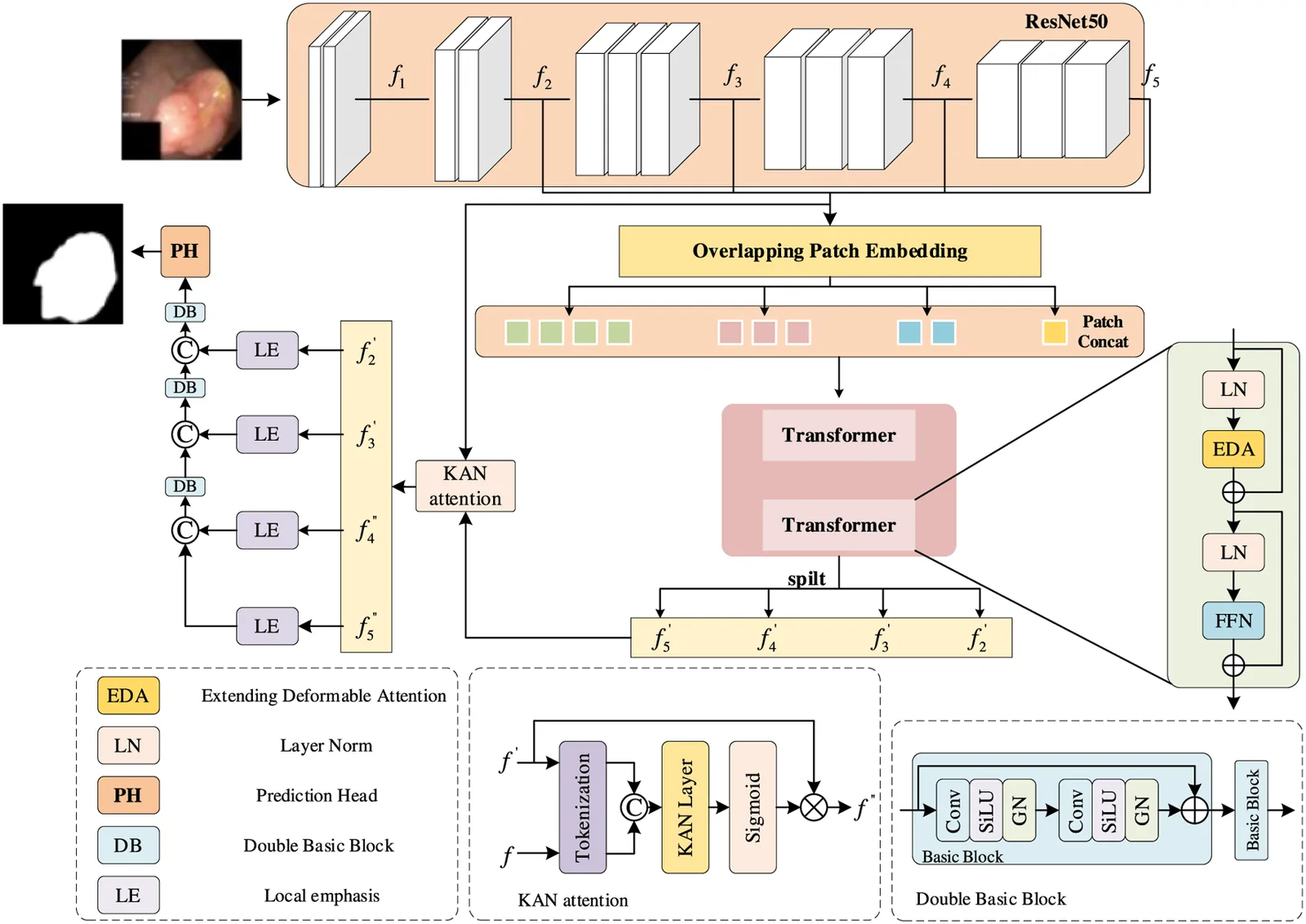
Overall architecture of TransCat, showing the CNN–Transformer encoder, Patch Concat, extended deformable attention, and KAN-based feature fusion.

The overall architecture of our proposed TransCat, as depicted in Figure 2, adopts an encoder-decoder hierarchical structure, employing a stacked approach to integrate the Transformer and CNNs as the encoder.

#### 3.1.1 Encoder

Given an input image $x\in R^{B\times H\times W\times C}$ , we first extract multi-scale feature maps using ResNet50 (He et al., 2016), as shown in Figure 2. These feature maps are then tokenized and concatenated through the proposed Patch Concat strategy before being fed into the Transformer branch. Compared with single-scale tokenization, Patch Concat preserves richer structural information for global modeling, but it also increases computational and memory costs because of the non-local nature of self-attention. To address this issue, we adopt deformable attention (Zhu et al., 2020) and further tailor it to the enlarged token set. The Transformer output is finally reshaped back into multi-scale feature maps that match the spatial scales of the encoder features.

#### 3.1.2 Decoder

The decoder takes the multi-scale features produced by the Transformer and ResNet50 branches as input. After spatial and channel alignment through a Local Emphasis module, the features are progressively refined via concatenation and Double Basic Blocks. The prediction head then generates the final segmentation map.

### 3.2 Patch concatenation

Dosovitskiy et al., 2020 proposed the ViT architecture, which accepts only single-scale input. To accommodate multi-scale features, Yang et al. (2023) first transformed features of different resolutions to the same scale through convolutional upsampling and then fused them by addition, enabling global modeling within the Transformer. However, this process may introduce interpolation noise, and additive fusion can discard complementary information across scales. We therefore propose a multi-scale feature input strategy called Patch Concat. Based on standard patch embedding, this strategy avoids explicit upsampling and replaces additive fusion with token concatenation, thereby preserving the original multi-scale information more faithfully.

**FIGURE 3:**
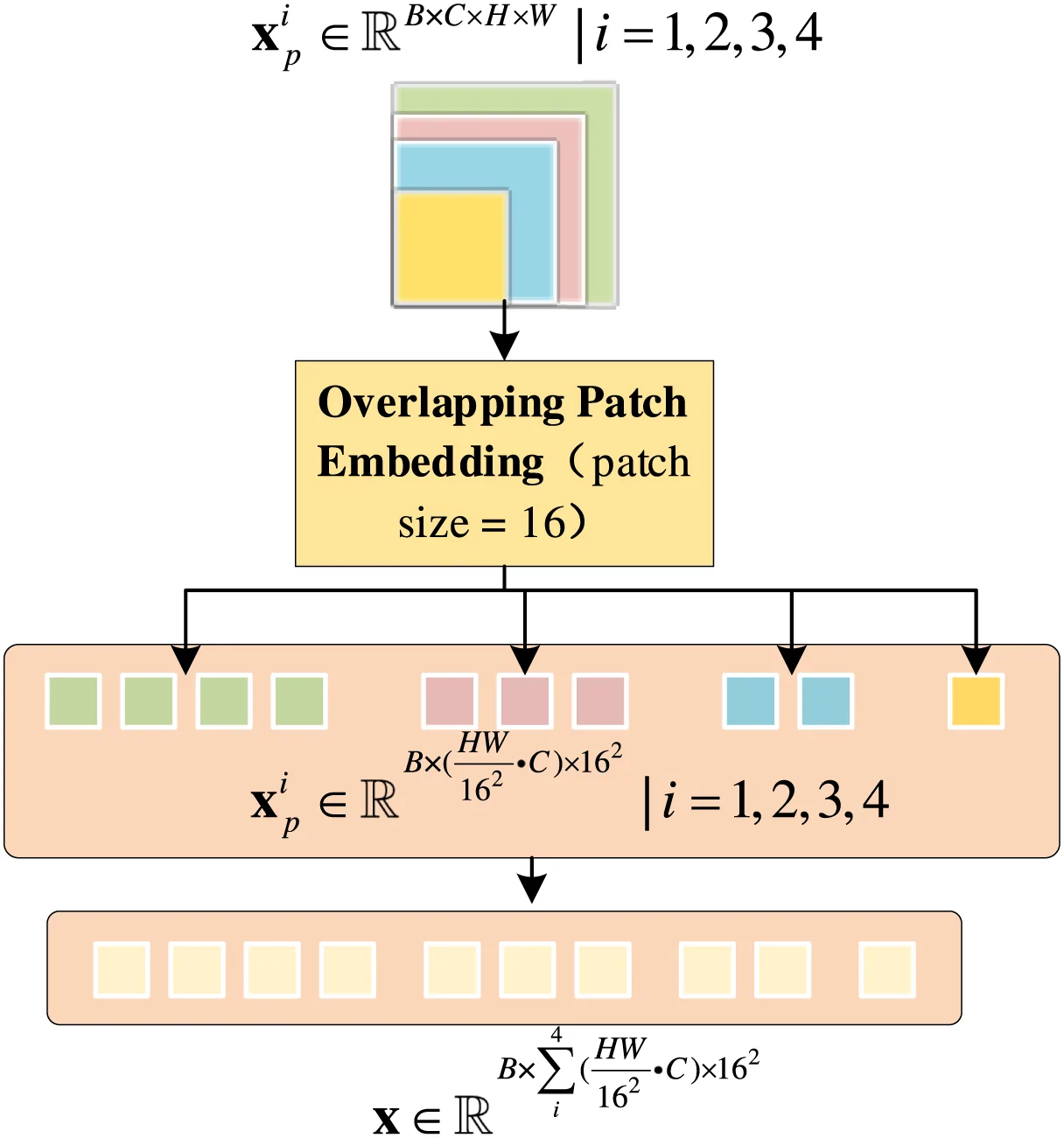
Patch Concat transforms multi-scale CNN feature maps into token sequences and concatenates them for joint global modeling in the Transformer branch.

As shown in Figure 3, let the i -th multi-scale feature map be denoted by $f^{i}\in R^{B\times H_{i}\times W_{i}\times C_{i}}$ , where i∈{1,2,3,4} . The overlap-preserving convolution described below first enriches each spatial location while retaining the feature-map size. Each channel is then partitioned into non-overlapping spatial windows of size P×P , with P=16 , and every window is flattened into a $P^{2}$ -dimensional token. Thus, the token sequence from scale i is given by Equation 1:

$$x_{p}^{i}\in R^{B\times \frac{H_{i}W_{i}C_{i}}{P^{2}}\times P^{2}}.$$ (1)

Because all scales share the same token dimension $P^{2}$ , their sequences can be concatenated along the token axis without requiring equal channel numbers:

$$x={\text{Concat}}_{i=1}^{4}\left(x_{p}^{i}\right)\in R^{B\times \left(\overset{4}{\underset{i=1}{\sum }}\frac{H_{i}W_{i}C_{i}}{P^{2}}\right)\times P^{2}}.$$ (2)

### 3.3 Deformable attention

#### 3.3.1 Revisiting deformable attention

Deformable attention is introduced to reduce the high memory cost of standard self-attention in Transformers. In deformable attention, each query attends only to a subset of sampling points around a reference point, which significantly reduces computational overhead. Given an input $x\in R^{B\times N\times C}$ , where q denotes a query element with content feature $z_{q}$ and a two-dimensional reference point $p_{q}$ , the deformable attention feature is computed as follows shown in Equation 3:

$$\text{DeformAttn}\left(z_{q},p_{q},x\right)=\overset{M}{\underset{m=1}{\sum }}W_{m}\left[\overset{K}{\underset{k=1}{\sum }}A_{\mathrm{mqk}}\cdot W_{m}x\left(p_{q}+\Delta p_{\mathrm{mqk}}\right)\right]$$ (3)

where m represents the attention head, k represents the sampling point, K represents the total number of sampling points, $A_{\mathrm{mqk}}$ and $\Delta p_{\mathrm{mqk}}$ respectively denote the attention weight and sampling offset of the $k^{th}$ sampling point, and $W_{m}$ represents the weight.

#### 3.3.2 Extending deformable attention for TransCat

Because patch concatenation substantially increases the number of input patches, using a fixed number of sampling points in deformable attention becomes less effective. Simply increasing the number of sampling points and their associated attention weights would introduce unnecessary computational cost. We therefore enhance deformable attention in the following three ways:

##### 3.3.2.1 Overlap-preserving feature embedding before patch partition

In medical image segmentation, lesions and anatomical regions often exhibit strong spatial continuity. We therefore apply a convolutional embedding with kernel size k=3 , stride s=1 , and padding p=1 before forming the P=16 spatial patches. For the i -th feature map, the output size is $H_{i}^{\text{out}}=\left\lfloor \frac{H_{i}+2p-k}{s}\right\rfloor +1$ and $W_{i}^{\text{out}}=\left\lfloor \frac{W_{i}+2p-k}{s}\right\rfloor +1$ . These settings give $H_{i}^{\text{out}}=H_{i}$ and $W_{i}^{\text{out}}=W_{i}$ , while each location incorporates its 3×3 neighborhood. The two sizes therefore refer to different operations: k=3 specifies the local overlap-preserving convolution, whereas P=16 specifies the subsequent patch partition used to construct the tokens in Equation 2.

##### 3.3.2 2 Attentive value identification

**FIGURE 4:**
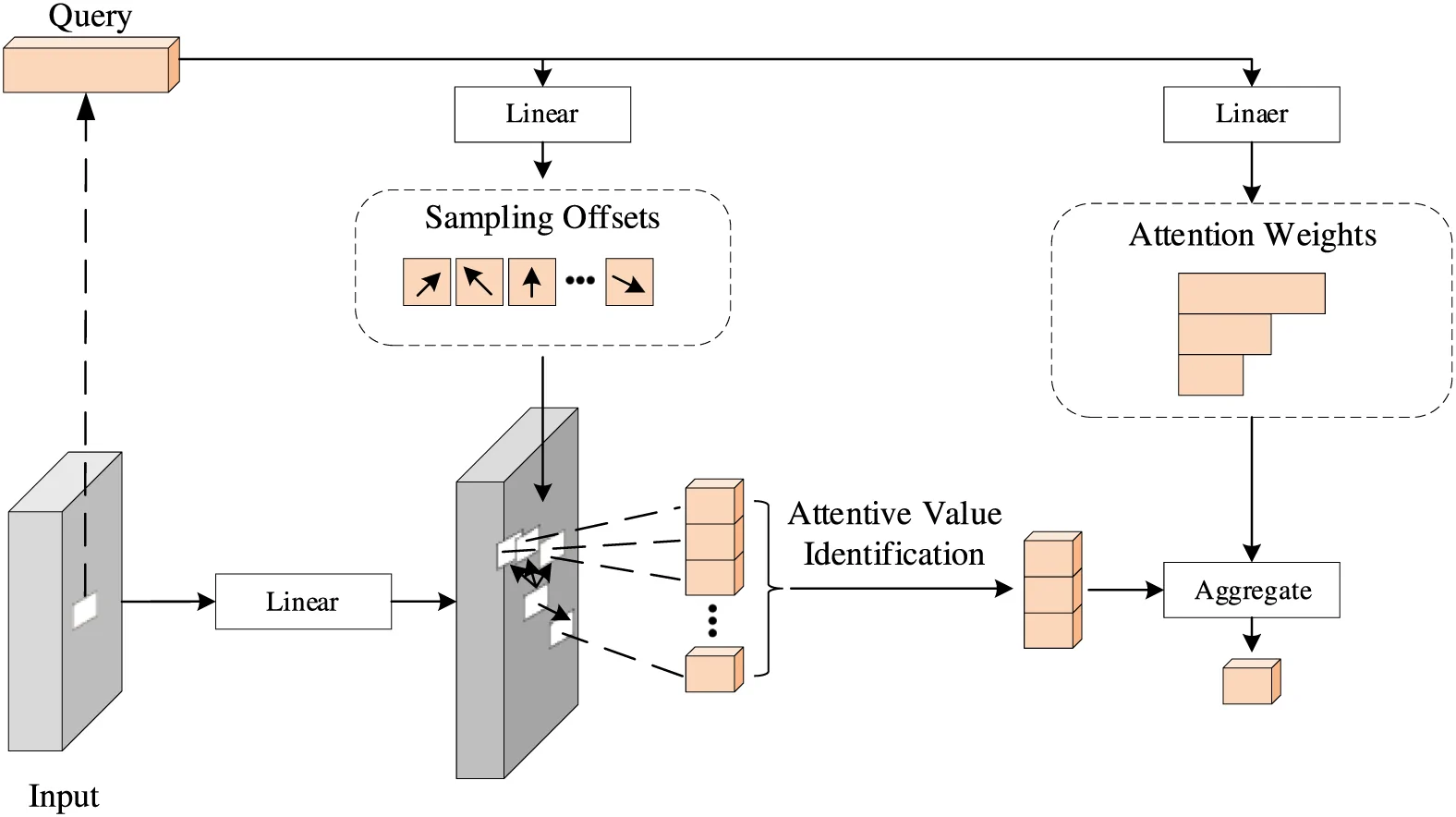
Extended deformable attention for a single head, illustrating enlarged candidate sampling and attentive value selection.

As illustrated in Figure 4, we increase the number of sampling points to generate a larger candidate set of keys and values. The dot-product response partially reflects the similarity between the query and each sampled key, that is, between the reference point and the sampling point. We therefore rank these responses and adopt a top- K strategy to select a fixed number of values for aggregation. Because sampling offsets and attention weights are independent, the number of sampling points can be increased while preserving compatibility with the existing attention weights.

##### 3.3.2 3 Removing the softmax operation

In standard self-attention, SoftMax normalizes all pairwise scores into a probability distribution, which is suitable when every value participates in a dense weighted sum (Vaswani et al., 2017). In the proposed extended deformable attention module, attentive value identification first forms a top- K subset from the enlarged sampled candidates; the selected values are then treated as a gated aggregation set rather than as a probability distribution over all candidate sampling locations. For selected responses $r\in R^{K}$ , applying SoftMax would impose $\sum_{k=1}^{K}\alpha_{k}=1$ and $\alpha_{k}\geq 0$ , which can compress relative magnitude differences among the selected responses. Removing the SoftMax operation keeps these selected responses less constrained before the following projection and normalization layers, allowing the module to retain relative response strength instead of forcing simplex-normalized weights. Related efficient attention studies also show that useful attention-style aggregation can be formulated without directly applying vanilla SoftMax over all token pairs (Katharopoulos et al., 2020; Choromanski et al., 2021). Therefore, our use of no SoftMax is a module-specific design choice for the top- K deformable aggregation path, and its convergence and usefulness are supported empirically by the ablation results rather than by a universal mathematical proof that SoftMax can always be removed.

**FIGURE 5:**
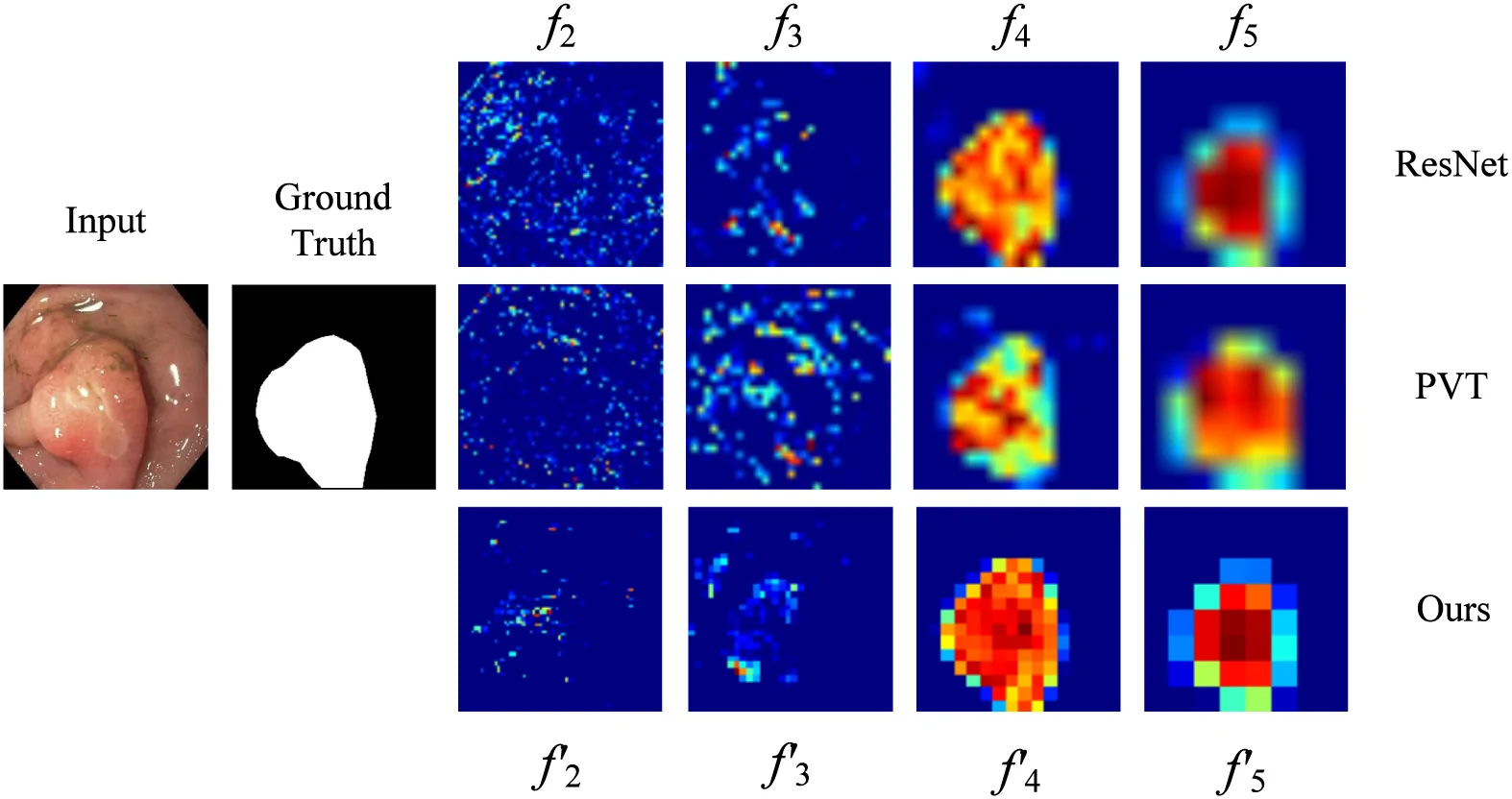
Attention heatmaps before and after multi-scale global modeling, together with a representative transformer baseline, illustrating the refinement of target-focused responses across scales.

To provide an intuitive demonstration of the effectiveness of the proposed multi-scale global modeling strategy, we visualize the attention heatmaps of multi-scale features before and after the Transformer module in Figure 5. From left to right, the figure shows the input image, the ground truth, and the corresponding attention heatmaps. The first row presents the heatmaps of multi-scale features extracted by ResNet. The third row shows the heatmaps after processing by the Transformer module in our model. For comparison, we also include a representative multi-scale Transformer baseline. Specifically, the second row shows the heatmaps generated by Pyramid Vision Transformer (PVT).

As shown in the figure, the heatmaps after multi-scale global modeling tend to concentrate more consistently on target-related regions and show reduced responses in some irrelevant background areas. At the same time, because deeper feature maps have lower spatial resolution, the visualization should not be interpreted as direct proof of sharper boundary delineation at every scale. Instead, the figure provides qualitative evidence that Patch Concat changes the distribution of attention toward task-relevant regions, complementing the quantitative results rather than replacing them.

### 3.4 KAN attention

The multi-scale features produced by the Transformer after patch concatenation already encode useful pixel-wise classification cues, but the local structural information preserved in the ResNet50 features remains valuable and should also be exploited. The lower-level feature maps contain more fine-grained structure as well as more background responses, whereas high-level features contain richer semantic information. We therefore use the Transformer outputs directly for lower-dimensional representations and apply the proposed KAN attention module to fuse the high-dimensional ResNet50 and Transformer outputs.

KANs (Liu et al., 2024) are motivated by the Kolmogorov-Arnold representation theorem (Kolmogorov, 1957) and use learnable univariate functions as part of their transformation layers. In this work, we do not use KAN as a replacement for the full segmentation backbone. Instead, we use it as a compact nonlinear mapping for estimating data-dependent fusion responses from paired CNN and Transformer features. This practical role is the basis for introducing KAN attention in TransCat.

Let $F_{c},F_{t}\in R^{B\times H\times W\times C}$ denote the spatially and channel-aligned high-level CNN and Transformer features, respectively. The tokenization operators $T_{c}$ and $T_{t}$ reshape the paired features into a common token representation, after which they are concatenated and passed through the KAN layer to produce unnormalized fusion responses, as defined in Equation 4:

$$R=\text{KAN}\;\left(\text{Concat}\;\left[T_{c}\left(F_{c}\right),T_{t}\left(F_{t}\right)\right]\right).$$ (4)

After reshaping R to the spatial layout of $F_{t}$ , a sigmoid function converts it into element-wise modulation weights A , and the fused output is given by Equation 5

$$A=\sigma (R),\;F_{\text{out}}=A⊙F_{t},$$ (5)

where ⊙ denotes element-wise multiplication. Thus, the CNN feature does not enter the output through direct addition; instead, it conditions the weights that selectively modulate the globally modeled Transformer feature. The KAN responses are fusion logits before sigmoid and learned modulation weights afterward, not pixel-class probabilities, calibrated confidence values, or direct evidence of clinical relevance.

## 4 Experiments

### 4.1 Datasets

To evaluate the proposed method, we consider eight benchmark datasets covering five segmentation tasks. CVC-300 and ETIS-LaribPolypDB are used only as additional unseen test sets in the cross-dataset polyp experiment.

- Kvasir-SEG (Jha et al., 2020): The Kvasir-SEG dataset includes 1,000 images with corresponding ground-truth annotations, and the image resolutions range from 720 × 576 to 1920 × 1072 pixels.
- CVC-ClinicDB (Vazquez et al., 2017): The CVC-ClinicDB consists of 612 endoscopic images, each annotated with segmentation masks for polyps and background.
- CVC-ColonDB (Bernal et al., 2012): The CVC-ColonDB dataset comprises 380 endoscopic images with corresponding polyp masks.
- CVC-300 (Vazquez et al., 2017): CVC-300 is a 60-image subset of the EndoScene test data and is used as an unseen polyp test set.
- ETIS-LaribPolypDB (Silva et al., 2014): This dataset contains 196 polyp images extracted from 34 colonoscopy sequences and is used only for external testing.
- Synapse (Landman et al., 2015): The Synapse dataset consists of abdominal CT images annotated for eight organs from 30 subjects, totaling 3,779 clinical CT slices. Each axial file contains between 85 and 198 slices of size 512 × 512 pixels.
- ISIC2018 (Tschandl et al., 2018): The ISIC2018 was released by the International Skin Imaging Collaboration (ISIC) and comprises 2,594 skin dermoscopy images, each accompanied by corresponding ground truth masks for lesion segmentation.
- 2018 Data Science Bowl (2018DSB) (Caicedo et al., 2019): This dataset was released for a Kaggle competition and contains 670 microscopy images with nucleus annotations.

### 4.2 Implementation details

The eight datasets are organized into five tasks: Kvasir segmentation, cross-dataset polyp segmentation, skin lesion segmentation, multi-organ segmentation, and nucleus segmentation. For the cross-dataset polyp task, following the commonly used setting in Fan et al. (2020), we randomly selected 90% of Kvasir-SEG (900 images) and approximately 90% of CVC-ClinicDB (551 images) for training. The remaining Kvasir-SEG images (100 images) and CVC-ClinicDB images (61 images) form the corresponding held-out test splits. The complete CVC-ColonDB (380 images), CVC-300 (60 images), and ETIS-LaribPolypDB (196 images) datasets are used as unseen external test sets and are not used for training or validation. No separate validation subset is listed for this cross-dataset protocol. Separately, the Kvasir single-dataset task uses only Kvasir-SEG with an 80:10:10 split to match prior single-dataset comparisons. For the Kvasir single-dataset and nucleus tasks, following Sanderson and Matuszewski (2022), we split each dataset into training, validation, and test sets with a ratio of 80:10:10. For the skin lesion task, following Xu et al. (2023), we used a 72:18:10 split. For the Synapse multi-organ task, we used the dataset partition listed in Table 1. All images were resized to 256 × 256 pixels. The detailed configuration is summarized in Table 1.

**TABLE 1:** The medical segmentation datasets used in our experiments. Kvasir-SEG appears in two settings: as part of the cross-dataset polyp task and as an independent Kvasir single-dataset task with an 80:10:10 split.

Task	Datasets	Images	Size	Train	Valid	Test
Polyp	Kvasir-SEG	1,000	256 × 256	900	—	100
	CVC-ClinicDB	612	256 × 256	551	—	61
	CVC-ColonDB	380	256 × 256	—	—	380
	CVC-300	60	256 × 256	—	—	60
	ETIS-LaribPolypDB	196	256 × 256	—	—	196
Kvasir single-dataset	Kvasir-SEG	1,000	256 × 256	800	100	100
Skin	ISIC2018	2,594	256 × 256	1868	467	259
Multi-organ	Synapse	3,779	256 × 256	2,211	—	1,568
Nucleus	2018DSB	670	256 × 256	536	67	67

Unless otherwise stated, the model was trained with a batch size of 16 and an initial learning rate of $1\times 10^{-4}$ . A learning-rate decay strategy was adopted: the learning rate was halved when the mDice score showed no improvement for 10 consecutive epochs, with a minimum learning rate of $1\times 10^{-6}$ . All experiments were implemented in PyTorch and conducted on a workstation equipped with an NVIDIA RTX 3090 GPU and 32 GB of RAM.

### 4.3 Evaluation results

We use four evaluation metrics, namely, Dice, mIoU, Recall, and Precision, to assess segmentation performance. These metrics are defined in Equations 6–9 as follows:

$$\text{Dice}=\frac{2TP}{2TP+FN+FP}$$ (6)

$$\text{mIoU}=\frac{1}{C}\overset{C}{\underset{c=1}{\sum }}\frac{TP_{c}}{TP_{c}+FP_{c}+FN_{c}}$$ (7)

$$\text{Precision}=\frac{TP}{TP+FP}$$ (8)

$$\text{Recall}=\frac{TP}{TP+FN}$$ (9)

Here, true positive (TP) denotes the number of pixels correctly segmented as lesion or target regions; true negative (TN) denotes the number of pixels correctly classified as background; false positive (FP) denotes the number of background pixels incorrectly classified as target pixels; and false negative (FN) denotes the number of target pixels incorrectly classified as background. In the mIoU formula, C is the number of classes.

For the polyp task, we additionally report $F_{\beta }^{\omega }$ , $S_{\alpha }$ , and $mE_{\xi }$ , as defined in Equations 10–12:

$$F_{\beta }^{\omega }=\frac{\left(1+\beta^{2}\right){\text{Precision}}^{\omega }\times {\text{Recall}}^{\omega }}{\beta^{2}{\text{Precision}}^{\omega }+{\text{Recall}}^{\omega }}$$ (10)

$$S_{\alpha }=\alpha \times S_{o}+\left(1-\alpha \right)\times S_{r}$$ (11)

$$E_{\xi }=\frac{1}{W\times H}\overset{W}{\underset{x=1}{\sum }}\overset{H}{\underset{y=1}{\sum }}\phi s\left(x,y\right)$$ (12)

where β is set to 1, and ω incorporates neighborhood information. Detailed derivations can be found in Margolin et al. (2014). Here, $S_{o}$ is the object-aware similarity term, $S_{r}$ is the region-aware similarity term, and α is empirically set to 0.5. In addition, W and H denote the image dimensions, and ϕs represents an enhanced alignment matrix that captures both pixel-level matching and image-level statistics of a binary map. Except for MAE, larger values indicate better segmentation performance. The MAE metric is defined in Equation 13:

$$\text{MAE}=\frac{1}{W\times H}\overset{W}{\underset{x=1}{\sum }}\overset{H}{\underset{y=1}{\sum }}|G\left(x,y\right)-S\left(x,y\right)|$$ (13)

where $S\in {\left[0,1\right]}^{W\times H}$ denotes the predicted map and $G\in {\left[0,1\right]}^{W\times H}$ denotes the ground-truth mask. Smaller MAE values indicate better segmentation quality.

Overall, the quantitative results show a mixed but generally competitive pattern. The clearest advantage appears in the cross-dataset polyp experiment on the unseen CVC-ColonDB dataset, whereas the gains on Kvasir-SEG, ISIC2018, Synapse, and 2018DSB are more modest and should be interpreted cautiously because the comparison protocols are not fully unified.

#### 4.3.1 Quantitative comparison across tasks

For the cross-dataset polyp setting, TransCat is compared with representative methods jointly trained on Kvasir-SEG and CVC-ClinicDB. As shown in Table 2, TransCat remains competitive on Kvasir-SEG and CVC-ClinicDB, while its clearest advantage appears on the unseen CVC-ColonDB test set, where it obtains the strongest reported mDice, mIoU, $F_{\beta }^{\omega }$ , $mE_{\xi }$ , and MAE values in this comparison. Table 3 further reports case-level bootstrap confidence intervals for the TransCat polyp results, which quantify test-set variability under the fixed evaluation splits rather than variability across repeated training seeds.

**TABLE 2:** Quantitative comparison on Kvasir-SEG, CVC-ClinicDB, and CVC-ColonDB.

Dataset	Methods	mDice	mIoU	$F_{\beta }^{\omega }$	$S_{\alpha }$	$mE_{\xi }$	MAE
Kvasir	UNet (Ronneberger et al., 2015)	0.818	0.746	0.794	0.858	0.881	0.055
	PraNet (Fan et al., 2020)	0.898	0.840	0.885	0.915	0.944	0.030
	ColonSegNet (Jha et al., 2021)	0.833	0.740	0.815	0.847	0.905	0.053
	ColonFormer-L (Duc et al., 2022)	0.916	0.865	0.908	0.928	0.957	0.026
	MEGANet (Bui et al., 2024)	0.913	0.863	0.907	0.918	0.955	0.025
	PVT-cascade (Rahman and Marculescu, 2023)	0.911	0.863	0.906	0.919	0.961	0.023
	CSCA U-Net (Shu et al., 2024)	0.903	0.846	0.890	0.918	0.946	0.031
	TransCat	0.915	0.859	0.902	0.916	0.951	0.027
CVC-ClinicDB	UNet (Ronneberger et al., 2015)	0.823	0.755	0.811	0.889	0.950	0.019
	PraNet (Fan et al., 2020)	0.899	0.849	0.896	0.936	0.975	0.009
	ColonSegNet (Jha et al., 2021)	0.805	0.705	0.776	0.847	0.925	0.041
	ColonFormer-L (Duc et al., 2022)	0.931	0.883	0.929	0.951	0.979	0.007
	MEGANet (Bui et al., 2024)	0.938	0.894	0.940	0.950	0.984	0.006
	PVT-cascade (Rahman and Marculescu, 2023)	0.919	0.872	0.918	0.936	0.969	0.013
	CSCA U-Net (Shu et al., 2024)	0.915	0.864	0.912	0.942	0.971	0.010
	TransCat	0.924	0.866	0.932	0.941	0.983	0.011
CVC-ColonDB	UNet (Ronneberger et al., 2015)	0.512	0.444	0.498	0.712	0.696	0.061
	PraNet (Fan et al., 2020)	0.712	0.640	0.699	0.820	0.847	0.043
	ColonSegNet (Jha et al., 2021)	0.676	0.569	0.640	0.774	0.835	0.046
	ColonFormer-L (Duc et al., 2022)	0.797	0.718	0.778	0.867	0.896	0.033
	MEGANet (Bui et al., 2024)	0.793	0.714	0.779	0.854	0.894	0.040
	PVT-cascade (Rahman and Marculescu, 2023)	0.789	0.710	0.779	0.855	0.896	0.031
	CSCA U-Net (Shu et al., 2024)	0.788	0.703	0.760	0.857	0.901	0.036
	TransCat	0.808	0.731	0.801	0.866	0.913	0.024

Red indicates the best performance and green indicates the second-best performance. Case-level bootstrap confidence intervals for the TransCat Dice and IoU results are reported separately in Table 3.

**TABLE 3:** Case-level bootstrap uncertainty estimates for TransCat on the polyp task. Values are reported as mean [95% CI] over the fixed test split. The intervals are obtained by resampling test cases with replacement and represent case-level test-set uncertainty, not variability across repeated training seeds.

Dataset	Cases	mDice	mIoU	Precision	Recall
Kvasir-SEG	100	0.9149 [0.8899, 0.9357]	0.8591 [0.8277, 0.8871]	0.9168 [0.8899, 0.9391]	0.9368 [0.9121, 0.9569]
CVC-ClinicDB	61	0.9237 [0.9026, 0.9404]	0.8657 [0.8363, 0.8907]	0.9226 [0.8937, 0.9455]	0.9372 [0.9178, 0.9548]
CVC-ColonDB	380	0.8075 [0.7815, 0.8316]	0.7306 [0.7040, 0.7559]	0.8156 [0.7901, 0.8404]	0.8492 [0.8233, 0.8736]
CVC-300	60	0.8532 [0.7966, 0.9011]	0.7845 [0.7227, 0.8365]	0.8087 [0.7457, 0.8619]	0.9656 [0.9549, 0.9748]
ETIS-LaribPolypDB	196	0.7505 [0.7067, 0.7915]	0.6720 [0.6304, 0.7122]	0.6932 [0.6484, 0.7354]	0.8607 [0.8169, 0.9014]

To provide an uncertainty estimate for the polyp task without claiming repeated training, we further computed per-case metrics from the available TransCat polyp checkpoint on the fixed test splits and estimated 95% confidence intervals using 10,000 case-level bootstrap resamples. As shown in Table 3, these intervals quantify test-set variability and should not be interpreted as standard deviations or confidence intervals across different random training seeds. For CVC-ClinicDB, the bootstrap calculation uses the 61-case held-out test split described in Table 1.

For the single-dataset Kvasir-SEG setting, Table 4 shows that TransCat reports the highest mDice and AR among the listed methods, reaching 0.9283 and 0.9365, respectively, while its mIoU and AP are not the highest. These results indicate that the proposed architecture is competitive in this setting rather than uniformly superior across all metrics.

**TABLE 4:** Performance comparison of different methods on the Kvasir dataset.

Methods	mDice	mIoU	AP	AR
U-Net (Ronneberger et al., 2015)	0.7821	0.8141	0.7241	0.8450
TransResUNet (Reza et al., 2020)	0.8884	0.8214	0.9106	0.9022
PraNet (Fan et al., 2020)	0.8942	0.8296	0.9126	0.9060
ColonSegNet (Jha et al., 2021)	0.8206	0.7239	0.8435	0.8496
MSRF-Net (Srivastava et al., 2021)	0.9217	0.8914	0.9666	0.9198
TGANet (Tomar et al., 2022a)	0.8982	0.8330	0.9123	0.9132
DCSAU-Net (Xu et al., 2023)	0.8885	0.8350	0.8950	0.8950
TransNetR (Jha et al., 2024)	0.8706	0.8016	0.9073	0.8843
DTAN (Zhao et al., 2024)	0.9039	0.8406	0.9201	0.9080
TransCat	0.9283	0.8774	0.9362	0.9365

Red indicates the best performance and green indicates the second-best performance.

For the Synapse multi-organ CT dataset, the quantitative results are summarized in Table 5. TransCat obtains an overall mDice of 80.35. Although this value is comparable to several listed baselines, the organ-wise DSC values are mixed, and the Synapse result should be regarded as a limitation of the current model rather than evidence of state-of-the-art performance on complex multi-organ CT segmentation. Domain-adaptive relational reasoning has also been investigated for 3D multi-organ segmentation (Fu et al., 2020).

**TABLE 5:** Performance comparison of different methods on the Synapse dataset.

Methods	mDice	DSC							
		Aor	Gal	LKid	RKid	Liv	Pan	Spl	Sto
V-Net (Milletari et al., 2016)	68.81	75.34	51.87	77.10	80.75	87.84	40.05	80.56	56.89
U-Net (Ronneberger et al., 2015)	76.85	89.07	69.72	77.77	68.60	93.43	53.98	86.67	75.58
R50-ViT (Dosovitskiy et al., 2020)	71.29	73.73	55.13	75.80	72.20	91.51	45.99	81.99	73.95
TransUNet (Chen et al., 2021)	77.48	87.23	63.13	81.87	77.02	94.08	55.86	85.08	75.62
UNETR (Hatamizadeh et al., 2022)	74.31	88.12	54.99	39.34	56.47	93.55	50.84	79.10	64.85
PraNet (Fan et al., 2020)	75.93	81.11	60.49	75.05	75.22	93.87	52.50	88.36	80.88
Adaptive t-vMF (Kato and Hotta, 2024)	78.27	85.64	62.10	79.47	78.54	91.47	52.25	81.68	73.65
Swin-UNet (Cao et al., 2022)	79.13	85.47	66.53	83.28	79.61	94.29	56.58	90.66	76.60
DA-TransUNet (Sun et al., 2024)	79.80	86.54	65.27	81.70	80.45	94.57	61.62	88.53	79.73
SelfReg-UNet (Zhu et al., 2024a)	80.34	88.74	71.78	85.32	80.71	93.80	62.22	84.78	75.39
TransCat	80.35	88.41	66.21	83.27	77.25	94.89	60.62	90.31	81.91

Red indicates the best performance and green indicates the second-best performance. The abbreviations are as follows: Aor, aorta; Gal, gallbladder; LKid, left kidney; RKid, right kidney; Liv, liver; Pan, pancreas; Spl, spleen; Sto, stomach.

For ISIC2018 skin lesion segmentation, Table 6 shows that TransCat reports the highest mDice in this comparison and remains competitive on the remaining available metrics, although some baselines do not report all metrics. These results suggest that the proposed framework is effective for skin lesion segmentation under the reported protocol.

**TABLE 6:** Performance comparison of different methods on the ISIC2018 dataset.

Methods	mDice	mIoU	AP	AR
U-Net (Ronneberger et al., 2015)	0.8524	0.7703	0.8843	0.8380
Attn U-Net (Oktay et al., 2018)	0.8448	0.7632	0.9042	0.8390
PraNet (Fan et al., 2020)	0.9117	0.8457	0.9120	0.9167
FANet (Tomar et al., 2022b)	0.8731	0.8023	0.9235	0.8650
PVT-GCASCADE (Rahman and Marculescu, 2024)	0.9151	0.8653	_	_
TransNetR (Jha et al., 2024)	0.8910	0.8215	0.8929	0.9196
ADF-Net (Huang et al., 2024)	0.9082	_	0.9670	0.9234
EMCAD (Rahman et al., 2024)	0.9096	_	_	_
TransCat	0.9189	0.8568	0.9334	0.9198

Red indicates the best performance and green indicates the second-best performance.

For the 2018DSB nucleus segmentation task, Table 7 shows that TransCat obtains the highest mDice and mIoU values among the listed methods while maintaining competitive AP and AR scores. This indicates favorable performance for fine-grained nucleus segmentation in the reported comparison, but it should still be interpreted with caution because the compared results are collected from different publications and protocols.

**TABLE 7:** Quantitative comparison on the 2018DSB dataset.

Methods	mDice	mIoU	AP	AR
U-Net (Ronneberger et al., 2015)	0.7779	0.6369	0.8823	0.6956
Attn U-Net (Oktay et al., 2018)	0.7993	0.6721	0.8824	0.7305
TransUNet (Chen et al., 2021)	0.8868	0.7927	0.9191	0.8567
TransAttUnet (Chen et al., 2023)	0.9162	0.8498	0.9746	0.9185
TransNetR (Jha et al., 2024)	0.9173	0.8521	0.9232	0.9158
DCSAU-Net (Xu et al., 2023)	0.9141	0.8501	0.9141	0.9241
CFANet (Lin et al., 2024)	0.8813	0.7869	0.9116	0.8531
SECA-Net (Zhu et al., 2024b)	0.8890	0.8188	0.8910	0.8596
TransCat	0.9226	0.8606	0.9270	0.9224

Red indicates the best performance and green indicates the second-best performance.

#### 4.3.2 Qualitative visual comparison

After presenting the quantitative evidence, we provide qualitative visual comparisons as supporting interpretation. These visual examples are not used as standalone proof of superiority; instead, they help illustrate typical segmentation behaviors that are consistent with the numerical results reported above.

**FIGURE 6:**
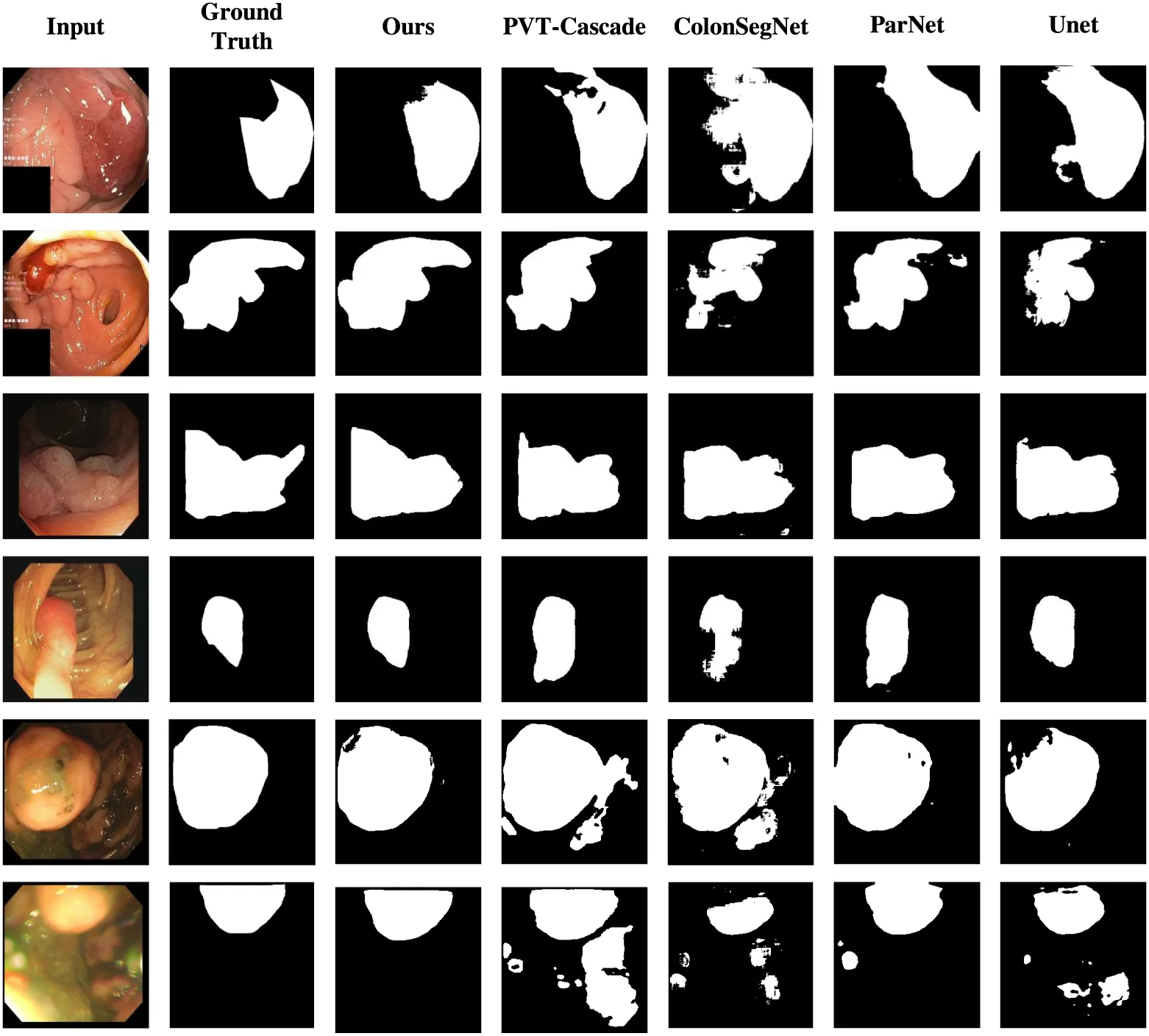
Qualitative comparison on polyp segmentation. The first two rows correspond to Kvasir-SEG, the next two rows to CVC-ClinicDB, and the last two rows to CVC-ColonDB. These examples provide visual context for the quantitative results rather than standalone evidence of generalization.

For polyp segmentation, Figure 6 provides examples from Kvasir-SEG, CVC-ClinicDB, and CVC-ColonDB. In the selected examples, TransCat produces relatively complete masks and fewer fragmented false positives in some challenging cases. These visual results are presented as supporting examples rather than evidence of general cross-dataset robustness.

**FIGURE 7:**
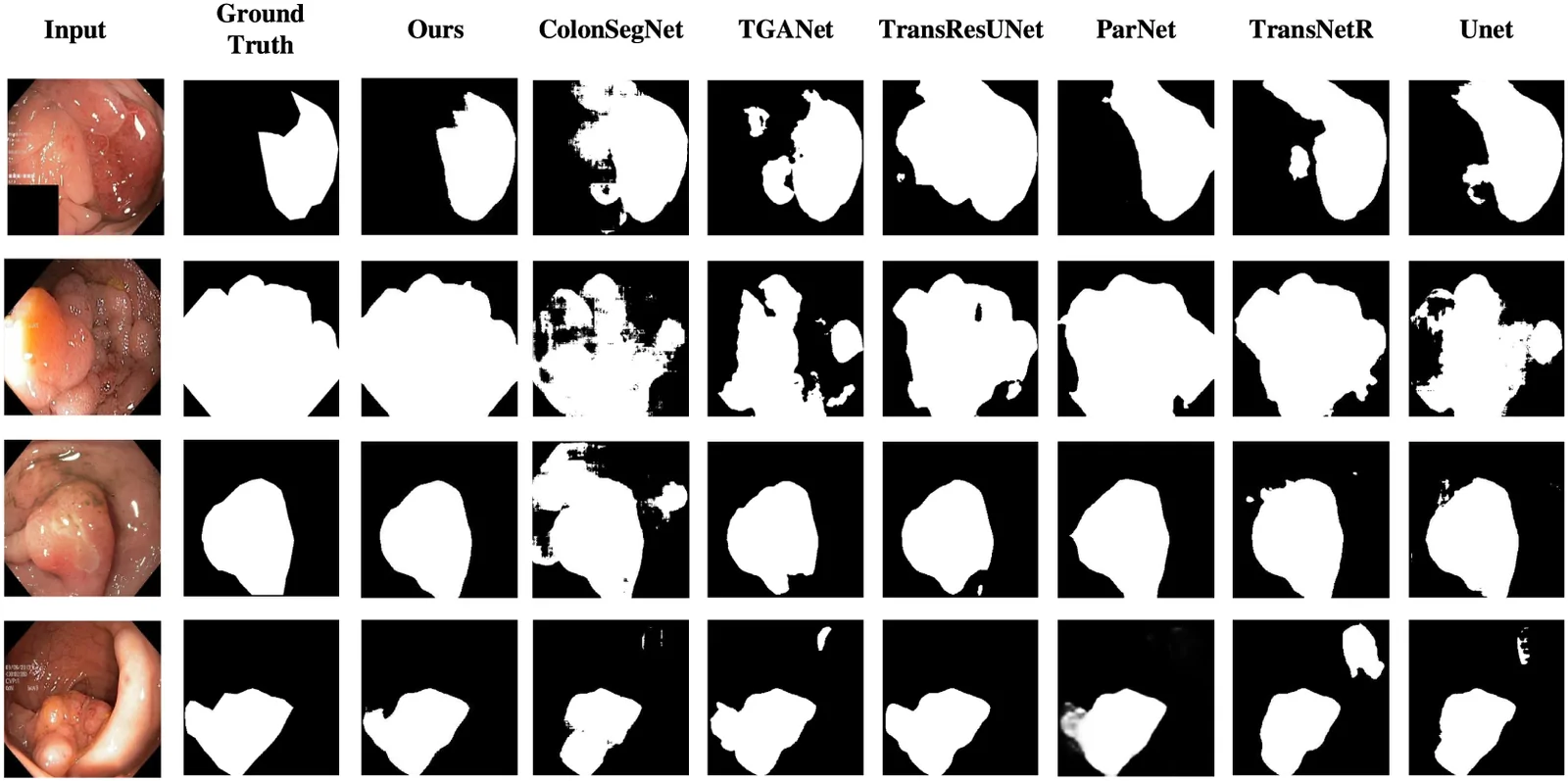
Qualitative comparison on the Kvasir-SEG dataset. The examples illustrate representative segmentation patterns and should be interpreted together with the quantitative metrics.

For the single-dataset Kvasir-SEG task, Figure 7 illustrates representative segmentation patterns. The examples are consistent with the design goal of coordinating local boundary extraction and global semantic reasoning, but they should be interpreted together with the quantitative results in Table 4.

**FIGURE 8:**
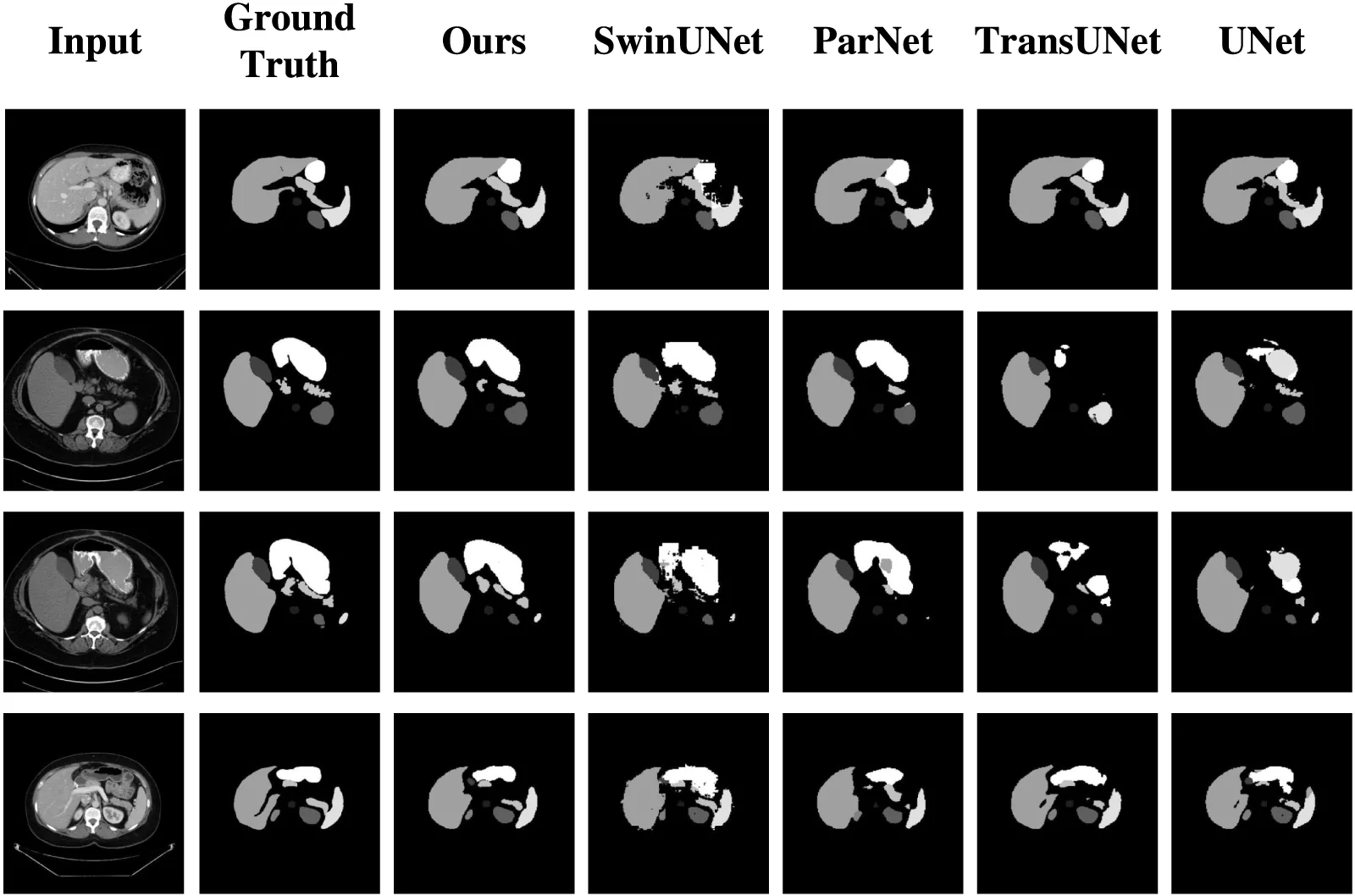
Qualitative comparison on the Synapse multi-organ CT dataset. The selected cases illustrate anatomical consistency visually, while strict ranking should rely on quantitative evaluation under unified protocols.

For the Synapse multi-organ CT dataset, Figure 8 is provided only as visual context for selected cases. Because the quantitative Synapse results in Table 5 are mixed, the qualitative examples should not be interpreted as proof of superiority on this dataset.

**FIGURE 9:**
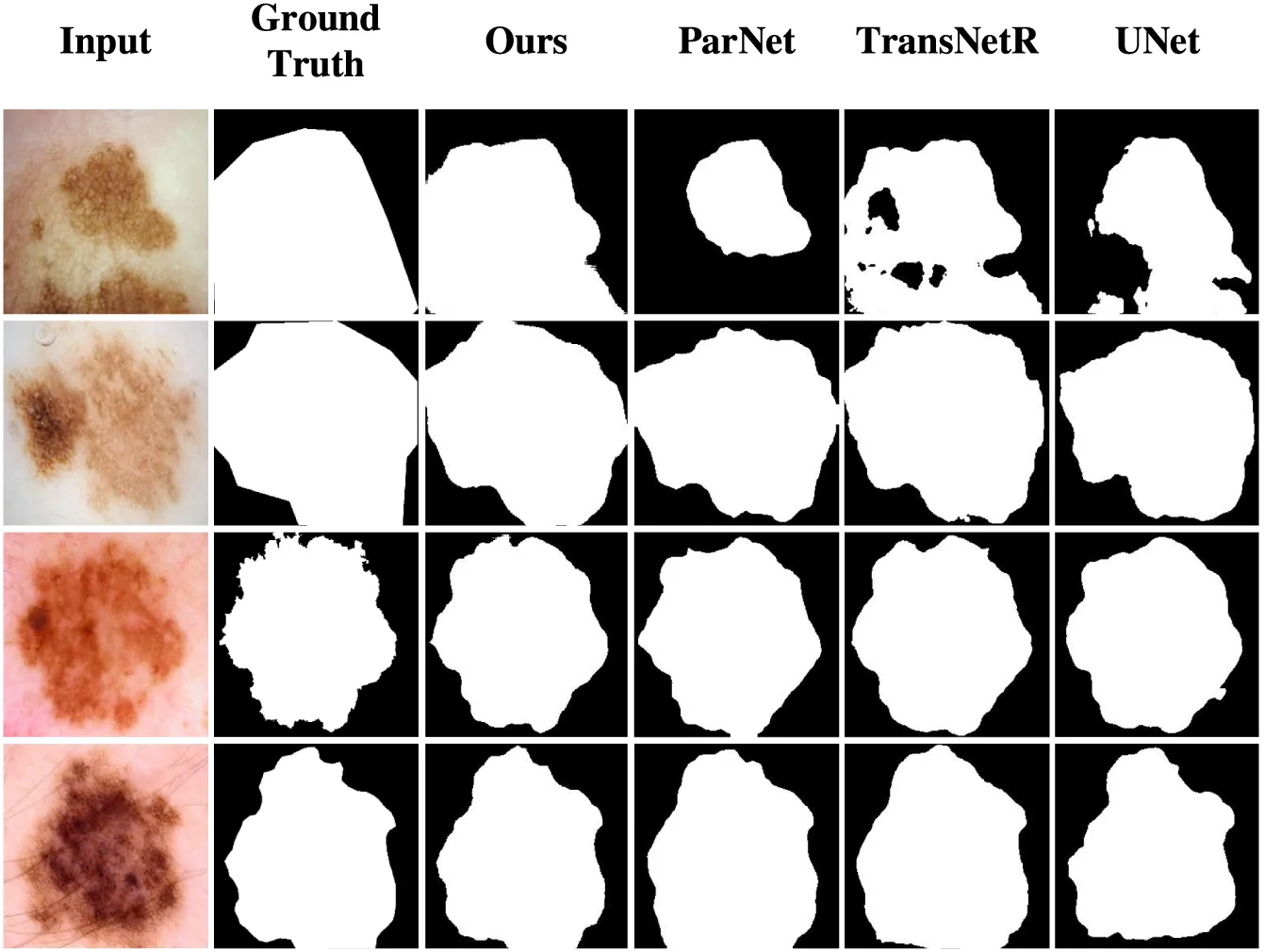
Qualitative comparison on the ISIC2018 skin lesion dataset. The selected examples show visual behavior under irregular lesion boundaries and low-contrast regions, but they are used only as supporting evidence.

For ISIC2018, Figure 9 illustrates visual behavior under irregular lesion boundaries and low-contrast regions. These examples support the interpretation that combining local detail with global context can be useful for skin lesion segmentation, while the primary evidence remains the quantitative comparison in Table 6.

**FIGURE 10:**
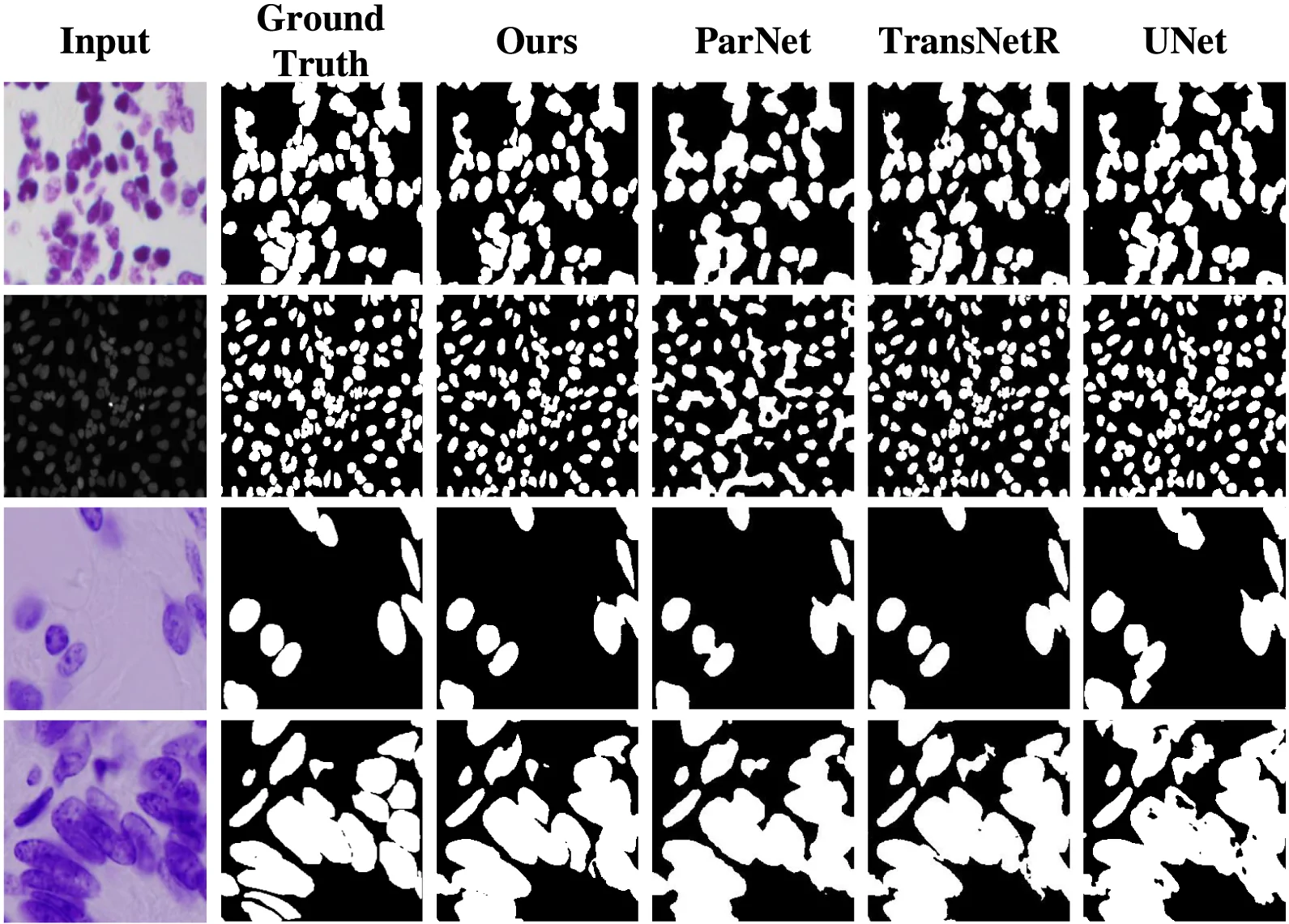
Qualitative comparison on the 2018 Data Science Bowl dataset. The visual examples are consistent with improved small-object localization in selected cases, but they do not replace the quantitative comparison.

For the 2018DSB nucleus task, Figure 10 provides examples related to small-object localization and adjacent-instance separation. The visual results are consistent with the favorable mDice and mIoU values in Table 7, but they are used only as complementary interpretation.

### 4.4 Computational efficiency

We evaluate computational efficiency from two theoretical perspectives: floating-point operations (FLOPs) and the number of parameters. These indicators describe model complexity but do not directly measure deployment behavior such as latency, frames per second, or peak GPU memory. Table 8 compares the models considered in this study. Among the listed values, DA-TransUNet has the highest FLOPs at 107.95G, whereas TransUNet has the largest parameter count at 96.07M. PVT-CASCADE has the lowest FLOPs at 5.98G, and PraNet has the fewest parameters at 32.55M. Because literature-reported complexity can depend on input size, operator counting, and implementation, this table should be interpreted as an approximate theoretical comparison rather than a hardware-level benchmark.

**TABLE 8:** Statistics of floating-point operations (FLOPs) and parameters (Params) of different models. These values describe theoretical model complexity and do not measure latency, FPS, or peak memory.

Methods	Params(M)	FLOPs(G)
PraNet (Fan et al., 2020)	32.55	13.08
UNETR (Hatamizadeh et al., 2022)	92.49	75.76
MEGANet (Bui et al., 2024)	44.19	28.68
CSCA U-net (Shu et al., 2024)	35.27	22.68
TransUNet (Chen et al., 2021)	96.07	68.48
DA-TransUNet (Sun et al., 2024)	84.52	107.95
TransAttUNet (Chen et al., 2023)	84.72	66.86
PVT-CASCADE (Rahman and Marculescu, 2023)	34.13	5.98
TransCat	63.95	56.17

TransCat requires 56.17G FLOPs and 63.95M parameters, so it should not be regarded as a lightweight model in absolute terms. Compared with several Transformer-heavy hybrids in Table 8, it provides a moderate accuracy-complexity trade-off, but it is heavier than lightweight CNN-based or efficient cascade models. Therefore, the extended deformable attention module should be interpreted as reducing the attention cost that would arise from direct dense attention over the enlarged token set, while the complete TransCat model remains computationally non-negligible.

### 4.5 Ablation experiments

In this section, we further evaluate the contributions of the proposed components through ablation studies on the extended deformable attention module and the KAN attention mechanism.

#### 4.5.1 Effectiveness of the extended deformable attention

As shown in Table 9, we conduct ablation experiments on the internal components of the proposed extended deformable attention module. The first row reports the performance of the baseline deformable attention, and the following rows show the incremental effects of the proposed modifications. The results indicate that each component contributes positively to the final performance.

**TABLE 9:** Ablation study of the proposed extended deformable attention on the Kvasir dataset. Only mDice is reported because the intermediate variants in this component ablation were selected using mDice and complete mIoU records were not available for all rows.

#	Configuration	mDice
1	Baseline deformable attention	0.8895
2	+ Overlapping patch embedding	0.8978
3	+ Removing softmax normalization	0.9035
4	+ Attentive value identification	0.9283

More specifically, overlapping patch embedding yields an immediate performance gain, suggesting that local neighborhood continuity remains important even when the downstream reasoning module is Transformer-based. Removing the softmax operation further improves the result in this setting, which empirically supports the use of a less constrained gated aggregation over selected top- K sampled values. The largest gain is obtained after introducing attentive value identification, showing that selecting informative sampled values is especially important once Patch Concat enlarges the token set. Table 9 reports mDice because it was the primary component-selection metric for this internal attention ablation; the corresponding mIoU values were not available for all intermediate variants.

The present ablation focuses on the internal components of the proposed attention module on Kvasir-SEG. A more exhaustive sensitivity analysis of patch size and the top- K value, preferably with repeated runs and FLOPs/Params for each setting, would provide a stricter assessment of these hyperparameters. Since such complete sensitivity results are not available for all settings, we report the current component-level ablation and discuss broader sensitivity testing as future work.

#### 4.5.2 Effectiveness of KAN attention

To examine the effect of KAN attention within the tested fusion choices, we compare it with two simple fusion strategies, namely, element-wise addition and concatenation, and two lightweight adaptive fusion baselines, namely, SE-based fusion and CBAM-based fusion (Woo et al., 2018). Cross-attention fusion is not included in this focused ablation because it introduces a substantially different interaction structure and computational cost, making it less directly comparable as a lightweight high-level fusion replacement. As shown in Table 10, KAN attention achieves an mDice of 0.9283 and an mIoU of 0.8774. Relative to feature concatenation, the strongest non-KAN alternative in this comparison, the absolute improvements are 0.0077 in mDice and 0.0122 in mIoU. Relative to using Transformer features alone, the improvements are 0.0101 and 0.0160, respectively.

**TABLE 10:** Comparison of feature fusion strategies on the Kvasir dataset. SE-based and CBAM-based fusion are added as lightweight adaptive fusion baselines to test whether the gain of KAN attention comes merely from adding a learnable fusion block.

#	Fusion strategy	mDice	mIoU
1	Transformer features only	0.9182	0.8614
2	Element-wise addition	0.9156	0.8569
3	Feature concatenation	0.9206	0.8652
4	SE-based adaptive fusion	0.9174	0.8630
5	CBAM-based adaptive fusion	0.9131	0.8560
6	KAN-based adaptive fusion	0.9283	0.8774

The comparison reveals three points. First, simply adding the CNN features does not guarantee improvement: element-wise addition produces lower mDice and mIoU than the Transformer-only variant, suggesting that unselective local responses can interfere with the globally modeled representation. Second, concatenation performs better than addition and the tested SE- and CBAM-based variants, suggesting that preserving complementary branch information is useful in this setting, but it still lacks explicit pixel-wise control over how that information modulates the Transformer features. Third, the higher scores obtained by KAN attention are consistent with the intended effect of learning a nonlinear, data-dependent fusion response from the paired features. The focused ISIC2018 ablation provides additional support: removing KAN attention and using feature concatenation reduces mDice from 0.9189 to 0.9129 and mIoU from 0.8568 to 0.8501. These improvements are meaningful within the tested settings, but they do not establish that KAN attention is superior to every adaptive fusion or cross-attention design.

**FIGURE 11:**
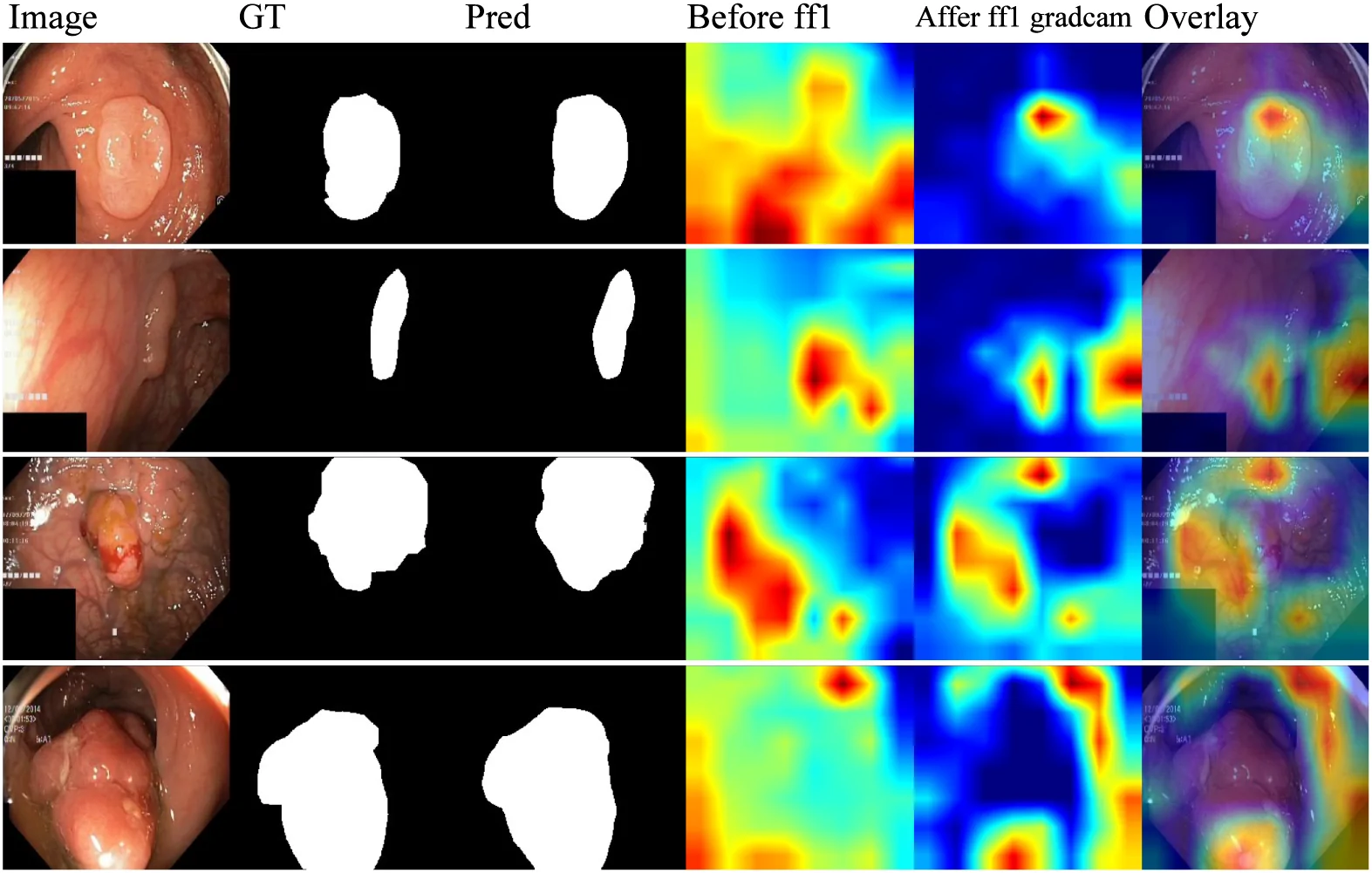
Grad-CAM visualization of the first high-level KAN fusion module on representative Kvasir-SEG test images. The figure is composed of four selected ff1 panels: sample_00_idx_0521, sample_01_idx_0319, sample_02_idx_0059, and sample_03_idx_0820. Each panel shows the original image, ground-truth mask, prediction, before-fusion activation, after-fusion Grad-CAM response, and the Grad-CAM overlay. The heatmaps provide qualitative evidence that the KAN-based fusion response tends to concentrate on polyp-related regions and suppress part of the irrelevant background response. These visualizations are intended as interpretive support and should be considered together with the quantitative ablation results.

To further interpret the behavior of KAN-based fusion, we visualize representative Kvasir-SEG test cases using Grad-CAM in Figure 11. In the selected cases, the post-fusion responses tend to become more concentrated on polyp-related regions while some background activations are reduced. This behavior is consistent with selective modulation of the Transformer features by paired CNN evidence. Nevertheless, Grad-CAM is resolution-dependent and provides only qualitative localization evidence; therefore, Table 10 and the additional ISIC2018 ablation remain the primary evidence for the impact of KAN attention.

#### 4.5.3 Additional component ablation on ISIC2018

To examine whether the component-level findings are limited to Kvasir-SEG, we further conducted a focused ablation study on the ISIC2018 skin lesion segmentation dataset. As shown in Table 11, removing Patch Concat reduces mDice from 0.9189 to 0.9094 and mIoU from 0.8568 to 0.8448, while replacing KAN-based fusion with feature concatenation reduces mDice to 0.9129 and mIoU to 0.8501. These results provide additional cross-dataset support for the contributions of Patch Concat and KAN attention under a different lesion segmentation setting. However, because this is a focused single-seed ablation on one additional dataset, we interpret it as supporting evidence rather than definitive proof of uniform component gains across all medical segmentation tasks.

**TABLE 11:** Additional component ablation on the ISIC2018 dataset. All variants use the fixed42 split and seed 2021. Params and FLOPs are theoretical complexity estimates for a 256 × 256 input and do not measure deployment latency or memory usage.

Variant	mDice	mIoU	AP	AR	Params (M)	FLOPs (G)
Full TransCat	0.9189	0.8568	0.9334	0.9198	63.95	56.17
W/o patch Concat	0.9094	0.8448	0.9121	0.9289	69.03	31.86
W/o KAN attention	0.9129	0.8501	0.9127	0.9339	48.06	52.26

### 4.6 Discussion

The proposed TransCat model shows its strongest evidence on the evaluated polyp, skin lesion, and nucleus segmentation tasks, but the magnitude of the gains varies by task and metric. The most notable result appears in the polyp experiments: although TransCat remains competitive on Kvasir-SEG and CVC-ClinicDB, its clearest advantage is observed on the unseen CVC-ColonDB test set. In contrast, the Synapse multi-organ CT result is comparatively weak and is treated as a limitation rather than as evidence of broad state-of-the-art performance.

This performance pattern is aligned with the design of the model. Patch Concat preserves multi-scale CNN evidence for global reasoning, allowing the Transformer to operate on richer structural cues than those available in conventional single-scale tokenization. The extended deformable attention mechanism then makes this strategy computationally feasible by focusing aggregation on informative sampled values. KAN attention subsequently acts on the high-level branch representations rather than on the full encoder. Its impact is to use the joint CNN–Transformer feature pair to determine spatially varying modulation weights, so that the contribution of local structural evidence varies according to the joint feature content instead of being merged uniformly. The Kvasir fusion ablation shows that this choice improves over Transformer-only features, addition, concatenation, SE, and CBAM in the tested configuration. The additional ISIC2018 ablation shows a smaller but consistent benefit over concatenation, indicating that the effect is not confined to one polyp split. The Grad-CAM examples further suggest reduced background response in selected cases, although they do not independently prove the mechanism.

The magnitude and scope of this impact should be interpreted cautiously. KAN attention increases the nonlinearity and parameterization of the fusion stage, and the observed improvement may reflect both its learnable functional form and the presence of adaptive spatial weighting. The present experiments do not isolate these two factors completely, nor do they compare against every possible cross-attention or dynamic gating formulation. In addition, the weaker Synapse result indicates that improved high-level fusion alone is insufficient to resolve all difficulties in multi-organ segmentation, where organ scale, class imbalance, and anatomical variability may require more task-specific modeling.

From an application perspective, these properties are valuable because reliable medical image segmentation should remain robust across datasets, acquisition settings, and anatomical variability. However, the weaker Synapse result indicates that the current architecture does not yet provide uniformly strong performance across all segmentation scenarios, especially complex multi-organ CT segmentation. Broader validation and task-specific refinement are still required before making strong claims about real-world deployment or modality-independent generalization.

Despite these strengths, the current study still has several limitations. First, the Synapse result is not sufficiently strong for multi-organ CT segmentation, indicating that the current TransCat design is more effective on the evaluated polyp, skin lesion, and nucleus segmentation tasks than on complex multi-organ anatomical segmentation. Second, patch concatenation and deformable attention introduce non-negligible computational overhead, and this study reports FLOPs and parameter counts rather than deployment-level latency, FPS, or peak memory. Third, the baseline tables contain literature-reported values compiled from original method papers and subsequent comparative studies rather than results reproduced under one implementation; a unified reimplementation with identical preprocessing, data splits, training schedules, losses, metric code, and post-processing would provide a stricter benchmark. Fourth, although we provide case-level bootstrap confidence intervals for the polyp task, the current results do not include full repeated multi-seed training runs or formal significance tests, so small numerical margins should be interpreted cautiously. Fifth, although we add a focused ISIC2018 component ablation for Patch Concat and KAN attention, broader multi-task ablation validation remains necessary before claiming that every module contributes equally across all modalities and datasets. Sixth, while KAN attention improves over the tested simple fusion strategies, this does not establish superiority over all adaptive fusion modules; its feature-level behavior and interpretability still require more detailed analysis, such as fusion-weight visualization across lesion boundaries and semantic regions.

## 5 Conclusion and future work

In this paper, we propose TransCat, a hybrid medical image segmentation framework designed to improve multi-scale global modeling and cross-branch feature fusion. By transforming CNN features into concatenated patch tokens, the model enables the Transformer to reason jointly over multiple scales. To address the resulting computational burden, we develop an extended deformable attention mechanism with attentive value identification. We further introduce KAN attention to adaptively fuse high-dimensional features from the CNN and Transformer branches. Under the reported protocols, the experimental results indicate that TransCat is competitive on several evaluated polyp, skin lesion, and nucleus segmentation benchmarks, with its clearest advantage appearing on the unseen CVC-ColonDB test set. However, its performance on the Synapse multi-organ CT dataset remains limited, so the results should not be interpreted as a strict proof of universal superiority across all medical segmentation tasks.

In future work, we plan to further optimize deformable attention so that the number of sampling points can adapt dynamically to the number of patches and to improve the model’s suitability for complex multi-organ CT segmentation. We also aim to perform more exhaustive patch-size and top- K sensitivity analyses, conduct full repeated multi-seed experiments with confidence intervals and statistical testing across all major tasks, compare KAN attention with additional adaptive fusion baselines beyond the tested SE- and CBAM-based variants, and report deployment-oriented metrics such as latency, FPS, and peak memory. In addition, future validation should include unified retraining of representative baselines where feasible, more systematic visualization of KAN-based fusion responses, and evaluation on more diverse imaging modalities and multi-center datasets.

## Data Availability

The original contributions presented in the study are included in the article/supplementary material, further inquiries can be directed to the corresponding author.
